# Role of ferroptosis in chronic kidney disease

**DOI:** 10.1186/s12964-023-01422-8

**Published:** 2024-02-12

**Authors:** Shiyang Li, Qiuxia Han, Chang Liu, Yixue Wang, Fengxun Liu, Shaokang Pan, Lihua Zuo, Dan Gao, Kai Chen, Qi Feng, Zhangsuo Liu, Dongwei Liu

**Affiliations:** 1 Traditional Chinese Medicine Integrated Department of Nephrology, The First Affiliated Hospital of Zhengzhou University, Zhengzhou, 450052 Henan People’s Republic of China; 2 Research Institute of Nephrology, Zhengzhou University, Zhengzhou, 450052 Henan People’s Republic of China; 3 Henan Province Research Center for Kidney Disease, Zhengzhou, 450052 Henan People’s Republic of China; 4 Key Laboratory of Precision Diagnosis and Treatment for Chronic Kidney Disease in Henan Province, Zhengzhou, 450052 Henan People’s Republic of China; 5 Department of Nephrology, Beijing Chao-Yang Hospital, Capital Medical University, Beijing, 100020 People’s Republic of China; 6 Department of Pharmacy, The First Affiliated Hospital of Zhengzhou University, Zhengzhou, 450052 Henan People’s Republic of China; 7 Kaifeng Renmin Hospital, Kaifeng, 475000 Henan People’s Republic of China

**Keywords:** Chronic kidney disease (CKD), Ferroptosis, Molecular mechanisms, Regulators, Treatment progress

## Abstract

Chronic kidney disease (CKD) has historically been a significant global health concern, profoundly impacting both life and well-being. In the process of CKD, with the gradual loss of renal function, the incidence of various life-threatening complications, such as cardiovascular diseases, cerebrovascular accident, infection and stroke, is also increasing rapidly. Unfortunately, existing treatments exhibit limited ability to halt the progression of kidney injury in CKD, emphasizing the urgent need to delve into the precise molecular mechanisms governing the occurrence and development of CKD while identifying novel therapeutic targets. Renal fibrosis, a typical pathological feature of CKD, plays a pivotal role in disrupting normal renal structures and the loss of renal function. Ferroptosis is a recently discovered iron-dependent form of cell death characterized by lipid peroxide accumulation. Ferroptosis has emerged as a potential key player in various diseases and the initiation of organ fibrosis. Substantial evidence suggests that ferroptosis may significantly contribute to the intricate interplay between CKD and its progression. This review comprehensively outlines the intricate relationship between CKD and ferroptosis in terms of iron metabolism and lipid peroxidation, and discusses the current landscape of pharmacological research on ferroptosis, shedding light on promising avenues for intervention. It further illustrates recent breakthroughs in ferroptosis-related regulatory mechanisms implicated in the progression of CKD, thereby providing new insights for CKD treatment.

Video Abstract

**Supplementary Information:**

The online version contains supplementary material available at 10.1186/s12964-023-01422-8.

## Introduction

Chronic kidney disease (CKD) represents a significant global public health challenge characterized by its high prevalence, limited awareness, unfavorable prognosis, and substantial medical costs. In 2012, the Kidney Disease Improving Global Outcomes (KDIGO) organization developed clinical practice guidelines defining CKD as abnormalities of kidney structure or function that persist for more than 3 months and have health concerns [1]. Globally, CKD affects 8 to 16% of the population [2], and is very closely associated with adverse clinical outcomes and an increased risk of all-cause mortality [3]. Moreover, CKD serves as a risk factor for cardiovascular disease, infections, strokes and other life-threatening conditions, underscoring its status as a global disease burden [4–6]. Presently, CKD management primarily revolves around mitigating cardiovascular risk, controlling blood pressure and glucose levels, avoiding nephrotoxic drugs, and treating diabetes and other primary diseases. Clinical treatments of CKD is mainly focused on symptomatic and supportive treatment, including inflammation inhibition, regulation of acid-base balance and electrolyte disorders, and enhancement of renal blood circulation [2]. While these measures can delay the progression of CKD to end-stage renal disease (ESRD), effective therapeutic options remain limited, particularly interventions capable of reversing CKD progression. Based on the abovementioned clinical issues, it is imperative to investigate the molecular mechanism underlying the onset and progression of CKD to identify novel therapeutic targets.

The pathology of CKD is characterized by progressive nephron loss, microvascular damage, metabolic alterations, oxidative stress, and inflammation, culminating in fibrosis [7, 8]. While a fibrotic matrix initially contributes to tissue repair, after injury, chronic and persistent kidney damage in the development of CKD leads to uncontrolled fibrotic matrix deposition, resulting in reduced blood supply, destruction of normal organ structure, and eventual glomerulosclerosis, renal tubular atrophy, and interstitial fibrosis [9, 10]. In addition, acute kidney injury (AKI) has an important impact on the occurrence and development of CKD. AKI is characterized by the damage and death of renal tubular cells. Generally, after renal tubular epithelial cell injury, surviving renal tubular epithelial cells undergo dedifferentiation and proliferation to repair the damage [11]. However, according to recent studies, if renal tubular epithelial cells are poorly repaired, damaged renal tubular epithelial cells will produce and secrete profibrotic factors, leading to proximal tubular atrophy and renal interstitial fibrosis [12, 13]. In conclusion, preventing and postponing the development of renal fibrosis is a critical step in the treatment and reversal of CKD.

Ferroptosis, a recently identified form of cell death initially proposed by Dixon et al. in 2012 [14], is an iron-dependent form of cell death characterized by the accumulation of lipid reactive oxygen species (ROS) [15]. Unlike the formation of autophagosome in autophagy or nuclear fragmentation in apoptosis, ferroptosis is distinguished by diminished mitochondrial size, increased mitochondrial membrane density, reduced or absent mitochondrial cristae, destroyed mitochondrial outer membrane and normal nuclei. However, it must be emphasized that ferroptosis lacks morphological distinctions from other forms of necrotic cell death [16]. Ferroptosis exerts a significant impact on the processes of various diseases, including tumors, neurodegenerative diseases, stroke, ischemia–reperfusion injury, cardiovascular and cerebrovascular diseases [15, 17]. Moreover, ferroptosis contributes to fibrosis in multiple organs, such as the lungs, heart, and liver [18–20]. Ferroptosis has also been shown to play an important role in renal fibrosis, which is the most pivotal pathological change in CKD. Studies have indicated targeting ferroptosis with specific drugs promotes adaptive cell repair and ameliorates fibrosis, suggesting drug intervention in ferroptosis as a potential avenue for preventing kidney fibrosis [21]. Although ferroptosis has gained prominence in recent years, iron’s potential nephrotoxic role was recognized as early as the 20th centur [22]. This review explores the molecular mechanisms of ferroptosis and its involvement in CKD development, providing novel insights for the prevention and treatment of CKD.

## Molecular mechanisms of ferroptosis

**Fig. 1:**
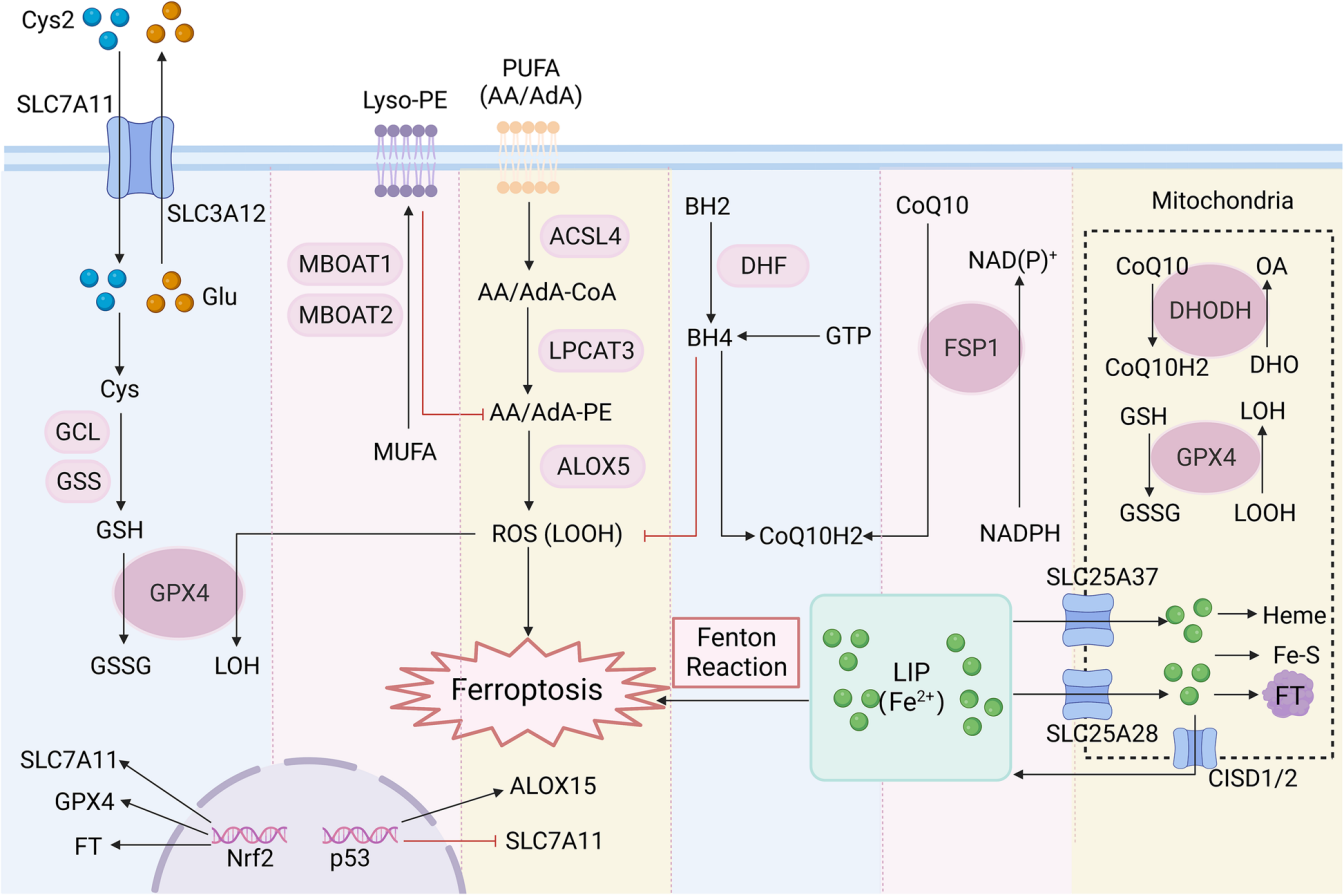
Mechanisms and key regulators of ferroptosis. Dysfunction of iron metabolism, lipid peroxidation and decreased antioxidant capacity are involved in the occurrence of ferroptosis. Key regulators in the antioxidant pathway, such as glutathione peroxidase 4 (GPX4), ferroptosis suppressor protein 1 (FSP1), tetrahydrobioptrin (BH4) and mitochondrial iron metabolism, play an important role in ferroptosis. The arrows represent promotion, and the red lines represent inhibition. Cys2, cystine; SLC7A11, solute carrier family 7 member 11; SLC3A12, solute carrier family 3 member 2; Glu, glutamic acid; Cys, cysteine; GCL, glutamic acid cysteine ligase; GSS, glutathione synthetase; GSH, glutathione; GSSG, Glutathione oxidized; Lyso-PE, lyso-phosphatidylethanolamine; MBOAT1, membrane bound O-acyltransferase domain containing 1; MBOAT2, membrane bound O-acyltransferase domain containing 2; MUFA, monounsaturated fatty acid; PUFA, polyunsaturated fatty acid; AA, arachidonoyl; AdA, adrenoyl; ACSL4, acyl-CoA synthesis long chain family member 4; LPCAT3, lysophosphatidylcholine acyltransferase 3; ALOX15, arachidonate 15-lipoxygenase; LOOH, lipid hydroperoxides; LOH, lipid alcohols; BH2, dihydrobiopterin; GTP, guanosine triphosphate; DHF, dihydrofolate; DHODH, dihydroorotate dehydrogenase; DHO, dihydroorotase; OA, orotic acid; SLC25A37, solute carrier family 25 member 37; SLC25A28, solute carrier family 25 member 28; FT, ferritin; CISD1/2, CDGSH iron sulfur domain 1/2; LIP, labile iron pool

Erastin and RSL3, two distinct small molecule inhibitors lacking structural similarities, were previously identified for their selective lethal impact on oncogenic RAS mutant cell lines. The traditional signs of apoptosis, such as mitochondrial cytochrome c release, caspase activation, and chromatin breakage, are notably absent in these dead cells [23, 24]. However, when these cells were co-treated with iron-chelating agents, the accumulation of ROS and cell death were inhibited. Thus, a new, nonapoptotic, iron-dependent form of cell death was inferred and given the name ferroptosis. The primary instigator of ferroptosis is the iron-dependent accumulation of ROS [14]. Since then, researchers have explored the molecular mechanism of ferroptosis and elaborated upon in detail below (Fig. 1).

### Disturbance of iron metabolism leads to ferroptosis

**Fig. 2:**
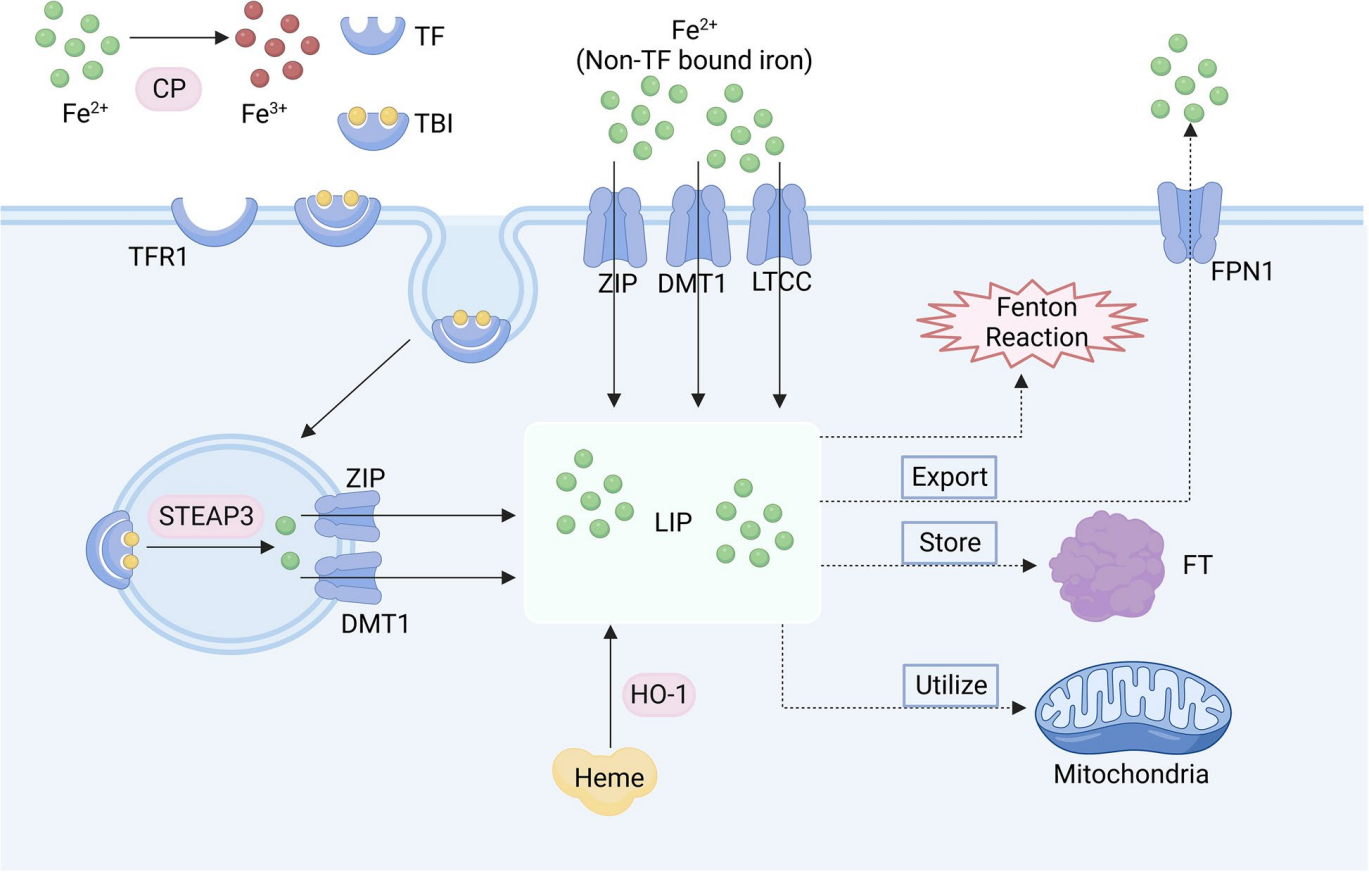
Metabolism of iron in cells. Iron metabolism is important for life. Fe^3+^ is mainly combined with TF to form TBI and then enters cells through endocytosis, while Fe^2+^ enters cells through DMT1, ZIP, LTCC and other pathways. Iron in LIP can be stored in FT or used by mitochondria. Iron output occurs mainly through FPN1

The core process driving ferroptosis is the disruption of iron metabolism. Iron ions possess the capability to capture and release electrons during the dynamic conversion between Fe^2+^ and Fe^3+^, sustaining ongoing redox reactions. As such, maintaining iron homeostasis is essential for normal life activities. Diseases such as organ fibrosis, myocardial diseases, endocrine diseases, and neurodegenerative diseases, have been found to be related to iron overload [25]. Under physiological conditions, Fe^2+^ in the blood is oxidized by ceruloplasmin (CP) to Fe^3+^, which then combines with transferrin (TF) to form transferrin-bound iron (TBI) [26]. TBI binds to transfer receptor 1 (TFR1) on the cell membrane, entering the cell through endocytosis. Following this, Fe^3+^ is transported to the LIP via divalent metal-ion transporter-1 (DMT1) or ZRT/IRT-like proteins (ZIP), eventually being utilized by the organism after being reduced to Fe^2+^ by the six-transmembrane epithelial antigen of prostate 3 (STEAP3) [25, 27]. However, TF’s binding sites exhibit limited affinity for iron ions. Iron ions not forming TBI predominantly bind with serum albumin and citric acid, constituting nontransferrin-bound iron (NTBI) [28]. NTBI enters cells through DMT1, ZRT/IRT-like proteins 8/14 (ZIP8/14), L-type calcium channel (LTCC), T-type calcium channel (TTCC), among others [29]. Iron from LIP is utilized by mitochondria, stored in FT, and exported through ferroportin 1 (FPN1), identified as the sole iron export route according to current research [30]. Reduced FPN and heightened iron input may contribute to renal tubular iron accumulation in CKD [31]. If Fe^2+^ is overloaded, the Fenton reaction will occur with LOOH to produce alkyl free radicals, instigating lipid peroxidation [30, 32]. Iron is also integral to enzymes associated with ferroptosis, with lipoxygenases (LOXs) utilizing Fe^2+^ as a cofactor to catalyze PUFA peroxidation [33]. Immunohistochemical methods were used to study the relationship between iron deposition, iron processing protein expression, and renal tubular injury in renal biopsies of CKD patients and healthy controls. Iron deposition occurred in the proximal tubules (PT) and distal tubules (DT) in 33% of CKD biopsies, but not in the control group [31]. Cultured human podocytes endocytose hemoglobin, induce oxidative damage, activate Nrf2, and induce the expression of Nrf2-associated antioxidant proteins heme oxygenase-1 (HO-1) and FT [34]. This intricate interplay underscores the critical importance of iron homeostasis in the context of ferroptosis (Fig. 2).

### Accumulation of LOOH leads to ferroptosis

LOOH is the executor of ferroptosis, and its cumulative buildup ultimately results in ferroptosis. The central and final step in ferroptosis involves the peroxidation of PUFAs within lipid membranes. ROS, byproducts of cellular aerobic metabolism, continuously undergo production, transformation, and consumption during the metabolic process of the body. Common ROS include superoxide (O2 -), hydroxyl radical (HO•), hydrogen peroxide (H_2_O_2_), and LOOH. Evidence from lipophilic antioxidant study substantiates LOOH as the primary culprit behind ferroptosis [35]. Both preexisting LOOH and LOO (.) are necessary for initiating lipid peroxidation. The existing LOOH is decomposed by Fe^2+^ and can be neutralized through reaction with Fe^2+^. However, when Fe^2+^ is oxidized to a certain extent, LOO (.) inhibition diminishes, marking the onset of lipid peroxidation [32]. During the process of ferroptosis, LOOH arises from the oxidation of phospholipids containing PUFAs. Due to the unstable carbon–carbon double bonds, PUFAs are more susceptible to lipid peroxidation than MUFAs, with arachidonoyl AA and AdA serving as their primary zymolytes [36]. Under the catalysis of ACSL4, AA and AdA form acyl Co-A derivatives [34]. Next, these derivatives are esterified by LPCAT3 to produce phosphatidylethanolamines (AA-PE and AdA-PE). ALOX15 then directly oxidizes AA-PE and AdA-PE to LOOH [36]. Studies demonstrate that exogenous MUFAs, such as exogenous oleic acid (OA) and palmitic oleic acid (POA), can replace PUFAs in the plasma membrane, reducing their sensitivity to oxidation, and thereby inhibiting ferroptosis caused by erastin and RSL3. This inhibition necessitates the activation of MUFAs by acyl-coenzyme A synthetase long-chain family member 3 (ACSL3) [37]. Oxidative stress is present in the early stages of CKD, contributing to renal function deterioration with increased antioxidant consumption and ROS generation [38, 39]. Compared with healthy controls, the levels of GPX4 and GSH were significantly decreased, and the levels of ROS and MDA were increased in the kidney tissues of CKD patients [40]. This underscores ferroptosis as a key pathological feature and a promising therapeutic target for CKD treatment.

### The system xc^−^/GPX4/GSH axis suppresses ferroptosis

Ferroptosis, characterized by uncontrolled membrane lipid peroxidation, hinges on the effective elimination of LOOH. The cystine/glutamate antiporter system (system Xc^−^)/GPX4/GSH axis plays a significant role in the elimination of lipid hydroperoxides. During aerobic metabolism, membrane components in cells inevitably produce LOOH, which is an important substrate for ferroptosis. To impede ferroptosis, GPX4 continuously converts LOOH into non-toxic lipid alcohols (LOH) in the presence of GSH [41, 42]. When LOOH is overloaded or GPX4 is absent or dysfunctional, Fe^2+^ reacts with LOOH, generating toxic lipid peroxidation, thereby triggering ferroptosis. GPX4 stands as a central inhibitor of ferroptosis, forming the cornerstone of anti-peroxidation defense [14]. System Xc^−^ is upstream of GPX4, belongs to the heterodimeric amino acid transporter (HAT) family, and consists of two subunits: the light chain subunit SLC7A11 and the heavy chain subunit SLC3A2 [43]. When affixed to the cell membrane, system Xc^−^ transfers intracellular Glu and extracellular Cys2 in a 1:1 ratio. Cys2 is exchanged with Glu through system Xc^−^ and enters the cell, where it is then reduced to Cys, which is then catalyzed by GCL and GSS to synthesize GSH [44]. GSH is a reducing substrate of GPX4 activity, which is essential for preventing and inhibiting ferroptosis. Previous studies have established that GSH protects cells and tissues from oxidative damage, and GSH deficiency is fatal to mammalian growth and development [45, 46]. Undoubtedly, the system Xc^−^/GPX4/GSH axis is the core regulator of ferroptosis.

### GPX4-independent ferroptosis inhibitory pathways

In 2019, Sebastian et al. identified FSP1, formerly known as apoptosis-inducing factor mitochondrial 2 (AIFM2), through an expression cloning approach, revealing its ability to prevent ferroptosis caused by GPX4 deletion. Irill et al. also identified that FSP1 can prevent ferroptosis through synthetic lethal CRISPR–Cas9 screening [47, 48]. Coenzyme Q10 (CoQ10) is a well-known lipophilic free radical that traps antioxidants. Functioning as an oxidoreductase, cardamoyl FSP1 catalyzes the regeneration and reduction of CoQ10 through NAD(P)H. This enzymatic process effectively prevents the transmission of lipid peroxides, thereby inhibiting ferroptosis. This is the first enzyme catalytic system capable of compensating for GPX4 deficiency. Kraft et al. uncovered a distinctive pathway independent of GPX4 that inhibits ferroptosis [49]. By performing a genome-wide activation screen, they discovered a group of genes antagonize ferroptosis, including guanosine triphosphate (GTP) cyclohydrolase-1 (GCH1) and its metabolic derivatives BH4/BH2. BH4 induces lipid reconstitution and inhibits ferroptosis by selectively preventing the depletion of phospholipids featuring two polyunsaturated acetyl chains. The regeneration of BH2 into BH4 is catalyzed by DHF [50]. Simultaneously, cells exhibiting elevated BH4 levels can alleviate oxidative damage by reducing CoQ10. This phenomenon might be attributed to the BH4-mediated conversion of phenylalanine to tyrosine, a process crucial for the production of 4-OH benzoate, a precursor in CoQ10 biosynthesis. In a recent discovery by Liang et al. using a whole-genome CRISPR activation screen, phospholipid-modifying enzymes MBOAT1 and MBOAT2 were identified as potent ferroptosis inhibitor factors. MBOAT1/2 selectively convert MUFAs to Lyso-PE, thereby inhibiting ferroptosis by reshaping the cellular phospholipid profiles. They demonstrated the ability to suppress ferroptosis independently of GPX4 or FSP1 [51]. The above are the pathways that have been found to inhibit ferroptosis independent of GPX4, while other undiscovered pathways that exist require further research.

### Mitochondrial dysfunction leads to ferroptosis

The role of mitochondria in ferroptosis stirred controversy, with varying perspectives on their participation in this intricate process. Mitochondria are bi-membrane organelles central to energy production, cell metabolism, and cell death and regeneration, also serving as primary generators of ROS [52]. During ferroptosis, the most obvious changes in mitochondria are morphological alterations, such as mitochondrial shrinkage, increased membrane density, and diminished or absent mitochondrial ridges [14]. Early observations by Dixon et al. indicated that cells lacking mitochondrial DNA exhibited a sensitivity to ferroptosis as cells with intact mitochondrial DNA [14]. However, conflicting findings emerged as Gaschler et al. proposed that mitochondria were not required to effectively inhibit ferroptosis [53]. However, Gao et al. demonstrated that cysteine deprivation led to mitochondrial membrane potential hyperpolarization and lipid peroxide accumulation. Notably, inhibition of the mitochondrial tricarboxylic acid (TCA) cycle or electron transfer chain (ETC) ameliorated these processes and inhibited ferroptosis. Importantly, these interventions had no effect on inhibiting GPX4-induced ferroptosis [54]. Other studies have also found that ETC complex inhibitors selectively inhibited ferroptosis caused by cysteine deprivation or erastin but proved ineffective against ferroptosis caused by the GPX4 inhibitor RSL3 [55, 56]. This discrepancy might be attributed to the ability of erastin to target voltage-dependent anion channels (VDACs) in mitochondria. Mito-TEMPO, a mitochondrion-targeted antioxidant, significantly rescued doxorubicin (DOX)-induced myocardial ferroptosis, substantiated that mitochondrial oxidative damage underlies ferroptosis-induced cardiac injury [57]. In cultured cardiomyocytes, overexpression of GPX4 or iron chelation targeting Fe^2+^ in mitochondria prevented DOX-induced ferroptosis, suggesting that DOX triggers ferroptosis in mitochondria [58]. Deactivation of DHODH can induce widespread mitochondrial lipid peroxidation and ferroptosis in low GPX4 cells. DHODH acts with mitochondrial GPX4, mediating the oxidation of dihydrolactate to OA and reducing CoQ10 to ubiquinol to inhibit ferroptosis in the inner mitochondrial membrane [59]. It is worth noting that Eikan et al. contested the experimental results of DHODH, posting that FSP1 played a pivotal role [60]. Mitochondrial iron dynamics are integral to ferroptosis modulation. Extracellular irons are absorbed by cells and enter mitochondria through SLC25A37 and SLC25A28. Mitochondrial Fe^2+^ generally severs three functions, including synthesis of heme and Fe-S clusters or storage in mitochondrial FT. In contrast, excessive mitochondrial Fe^2+^ may induce abnormal ROS production or enzyme activity [55]. Heme was identified as a ferroptosis trigger, with both cytoplasmic and mitochondrial heme oxygenase 1 (HMOX1) exerting control over this process [61]. Additionally, mitochondrial iron output proteins, such as CISD1 and CISD2, prevent ferroptosis by shielding mitochondria from lipid peroxidation [62]. A recent study found that cyclic GMP-AMP synthase (cGAS), anchored to the outer mitochondrial membrane, binds to dynamic-related protein 1 (DRP1) to facilitate its oligomerization, and protects liver cancer cells from ferroptosis [63]. As mentioned above, the involvement of mitochondria in ferroptosis has been demonstrated under certain conditions. However, the precise molecular mechanisms mediating this relationship require further investigation.

### Key regulators of ferroptosis

**Fig. 3:**
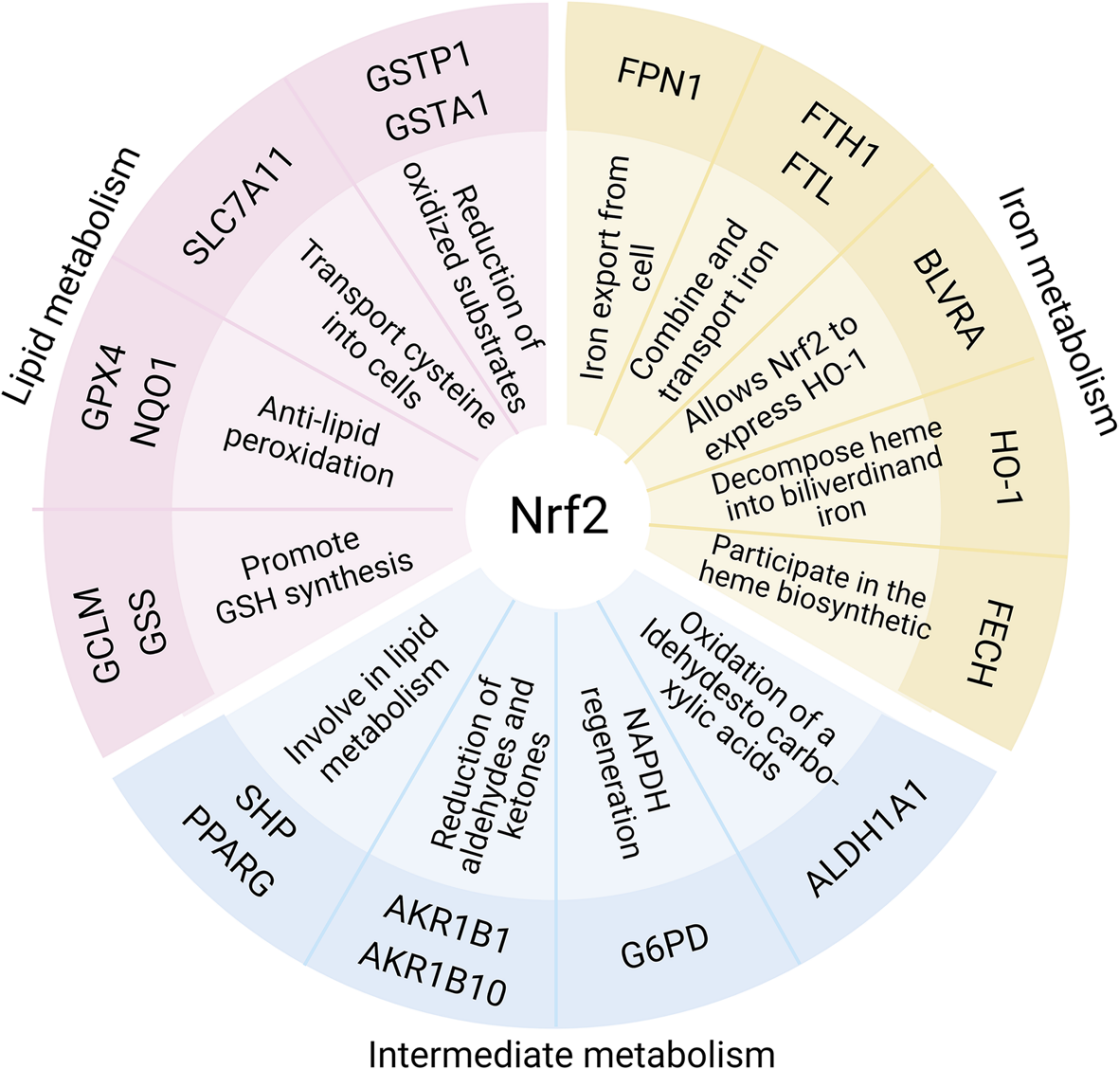
Target genes of Nrf2 involved in preventing ferroptosis. Nrf2 regulates ferroptosis mainly from three aspects: iron metabolism, lipid metabolism and intermediate metabolism. Many of the molecules involved in ferroptosis are target genes for Nrf2, which translocates to the nucleus and activates transcription of a number of antioxidant-related genes when cells are subjected to oxidative stress. GSTP1, glutathione S-transferase P1; GSTA1, glutathione S-transferase alpha 1; BLVRA, biliverdin reductase A; FECH, ferrochelatase; ALDH1A1, aldehyde dehydrogenase 1 family member A1; G6PD, glucose-6-phosphate dehydrogenase; AKR1B1, aldo-keto reductase family 1 member B1; AKR1B10, aldo-keto reductase family 1 member B10; SHP, small heterodimer partner; PPARG, peroxisome proliferator activated receptor gamma; NQO1, NAD(P)H quinone oxidoreductase 1

The intricate process of ferroptosis involves numerous genes and transcription factors, with a central role attributed to nuclear factor erythroid2-related factor 2 (Nrf2), drawing significant attention. Nrf2, a pivotal transcription factor responsible for maintaining cellular metabolism and redox balance, governs various molecules associated with ferroptosis, including FT light and heavy chains (FTL/FTH1), the system Xc^−^ subunit, the catalytic and regulatory subunits of glutamic acid cysteine ligase (GCLC/GCLM), as well as GPX4 [64, 65]. Typically, the E3 ubiquitin ligase complex comprised of Kelch-like ECH-associated protein 1-Cullin 3-Ring box 1 (KEAP1-CUL3-RBX1), S-phase kinase associated protein 1-Cullin 1-Rbx1/β-transducin repeat-containing protein (SCF/β-TrCP), and synonymin/Hrd1, continuously degrades Nrf2 proteasomes, maintaining Nrf2 at a low expression level [64]. However, under conditions of oxidative damage, Nrf2 ceases to be ubiquitinated and degraded, translocating to the nucleus to activate the transcription of numerous genes related to antioxidant defense [66]. Nrf2 mainly regulates ferroptosis across three dimensions: iron metabolism, lipid metabolism, and intermediate metabolism (Fig. 3) [64, 67, 68]. A study revealed that AKI repair was worse in males than in females. This difference was recently found to be due to sex differences in ferroptosis. Single-cell transcriptomic analysis identified the Nrf2 antioxidant protection pathway as a recovery mechanism against ferroptosis in females [69]. Further research found that deletion of Nrf2 led to apoferritin accumulation in the autophagosome, an elevated LIP, and heightened sensitivity to ferroptosis [70]. p53, another gene closely related to ferroptosis is the core of robust signaling networks. Activated by various upstream signaling pathways, p53 mediate downstream signaling pathways. Studies have shown that p53 can induce ferroptosis by inhibiting SLC7A11 transcription and upregulating ALOX15 [71, 72]. The p53 inhibitor pifithrin-α demonstrated efficacy in alleviating renal fibrosis [73]. Conversely, a constant and persistent basal level of p53 is believed to delay the onset of ferroptosis [74]. In addition to the abovementioned genes, more genes related to ferroptosis are shown in Table 1.

**Table 1:** Regulators related to ferroptosis

Classification	Gene	Regulatory mechanism	References
Drivers	p53	Inhibits SLC7A11 transcription and increases ALOX15 levels	[71, 72]
	Iron regulatory protein 2 (IRP2)	Regulates iron level by regulating the translation and stability of mRNA	[75]
	Nuclear receptor coactivator 4 (NCOA4)	Promotes ferritinophagy	[76]
	HMOX1	Catalytic degradation of heme and release of free iron	[77]
	NADPH oxidase 4 (NOX4)	Produces H_2_O_2_ and lipid peroxides	[78]
	ALOX12	Oxidates PUFA and induct p53	[79]
	Beclin 1 (BECN1)	Binds to SLC7A11 and block activity of Xc^−^	[80]
	Activating transcription factor 3 (ATF3)	Blocks Nrf2/KEAP1/Xc^−^ pathway	[81]
	LPCAT3	Generates C20:4 phospholipids	[82]
Suppressors	Nrf2	Promotes expression of FTL/FTH1, SLC7A11, GCLC/GCLM, FPN1and GPX4	[64, 67, 68]
	KEAP1	Stabilizes and regulate Nrf2	[83]
	Stearoyl-CoA desaturase (SCD)	Rate-limiting enzyme for monounsaturated fatty acid synthesis, leads to fatty acid desaturation	[84, 85]
	Cystathionine beta-synthase (CBS)	Decreases the methylation of ACSL4 protein and Degrades ACSL4 protein	[86]
	Ferritin mitochondrial (FTMT)	Stores iron in the mitochondria	[87]
	Rapamycin kinase (MTOR)	Regulation of ROS production	[88]
	Cadherin 1 (CDH1)	Regulation of iron and increase NAD(P)H/GSH levels	[89]
	Frataxin (FXN)	Regulation of iron homeostasis and mitochondrial function	[90]
	Heat shock protein beta-1 (HSPB1)	Reduces iron-mediated production of ROS	[91]
	Lipocalin 2 (LCN2)	Promotes the consumption of iron	[92]
	Phospholipase A2 group VI (PLA2G6)	Regulates P53 and hydrolyze phospholipid peroxide	[93, 94]
	Solute carrier family 40 member 1 (SLC40A1)	Inhibits iron transporter	[95]
	Signal transducer and Activator of transcription 3 (STAT3)	Inhibits expression of ACSL4	[96]
	Thioredoxin reductase (TXNRD)	Eliminates phospholipid hydroperoxides	[97]

### Agonists and inhibitors of ferroptosis

Ferroptosis presents a promising avenue for therapeutic intervention, and beyond the realm of genetic regulators, the identification of various small molecules associated with ferroptosis has opened up new possibilities. These molecules include both ferroptosis inducers and inhibitors, which showcase substantia therapeutic potential across a spectrum of disease. Under conditions characterized by abnormal cell proliferation, inducing ferroptosis proves advantageous in promoting the demise of malignant cells. Conversely, in other diseases where ferroptosis is detrimental, ferroptosis inhibitors can impede lipid peroxidation and ferroptosis related pathways, thereby delaying disease progression. The exploration of these small molecules targeting ferroptosis provides valuable insights into the potential treatment of CKD. Several common inducers and inhibitors related to ferroptosis are shown in Table 2.

**Table 2:** Agonists and inhibitors of ferroptosis

Classification	Reagents	Targets	References
Agonists	Erastin	System Xc^−^ (direct), VDAC2/3 (direct), p53 (enhances ferroptosis by activating p53)	[23, 98]
	RSL3	GPX4 (direct)	[14]
	Sulfasalazine	System Xc^−^ (a novel, potent inhibitor of System Xc^−^)	[99]
	Interleukin-6	System Xc^−^ (activates System Xc^−^ via the JAK2/STAT3 pathway)	[100]
	Sorafenib	System Xc^−^ (Inhibit System Xc^−^ by upstream)	[101]
	(R)-DCPP	GSH (unknown), GPX4 (unknown)	[102]
	Acetaminophen	GSH (direct)	[103]
	Cisplatin	GSH (direct)	[104]
	FINO2	GPX4 (indirect), Fe^2+^ (direct)	[105]
	Lipopolysaccharide	SLC7A11 (unknown), GPX4 (unknown)	[106]
	Legumain	GPX4 (promotes chaperone-mediated autophagy of GPX4)	[107]
	DPI2	GSH (unknown)	[41]
	Dexamethasone	GSH (indirect)	[108]
	Siramesine	FPN (unknown), FT (unknown)	[109]
	Dihydroartemisinin	FT (degradation of ferritin in an autophagy-independent manner)	[110]
	FIN56	GPX4, CoQ10 (GPX4 degradation and squalene synthase activation inhibit coenzyme Q10 through acetyl-CoA carboxylase activity)	[111]
	Manganese	ROS (direct), Lipid peroxidation (direct)	[112]
Inhibitors	Fer-1	ROS (direct)	[113]
	SRS 11–92	ROS (direct)	[114]
	Vitamin E	ROS (direct)	[115]
	Nuciferine	ROS (direct)	[116]
	Liproxstatin-1	ROS (direct)	[117]
	Irisin	Nrf2 (unknown), GPX4 (unknown)	[118]
	Platycodin D	GPX4 (unknown) regulates ferroptosis through GPX4	[119]
	Ginkgolide B	GPX4 (inhibiting the ubiquitination of GPX4.)	[120]
	Canagliflozin	system Xc^−^ (unknown), GPX4 (unknown), GSH (unknown)	[121]
	BRD4770	System Xc^−^-GPX4 (unknown), FSP1-CoQ10 (unknown), GCH1-BH4 (unknown)	[122]
	Quercetin	ROS (unknown), GSH (unknown)	[123]
	Hydropersulfides	ROS (direct)	[124]
	Ginsenoside Rg1	GPX4 (unknown), GSH (unknown), FSP1 (unknown) its antiferroptosis activity was dependent on FSP1	[125]
	MCTR1	Nrf2 (suppresses ferroptosis the Nrf2 signaling)	[126]
	Entacapone	Nrf2 (affects the p62-KEAP1-NRF2 pathway, thereby upregulatin NRF2)	[127]
	Astragaloside-IV	Nrf2 (inhibited miR-138-5p expression, subsequently increasing Sirt1/Nrf2 activity)	[128]
	Forsythoside A	Nrf2 (unknown), GPX4 (unknown) Nrf2/GPX4 axis plays a key role	[129]
	Rosiglitazone	ACSL4 (direct)	[130]
	Pioglitazone	ACSL4 (direct)	[130]
	Troglitazone	ACSL4 (direct)	[130]

## Ferroptosis and CKD

Decreased renal function often results in reduced iron bioavailability, cause iron deficiency in circulation, yet renal cells are vulnerable to iron overload [131, 132]. Iron deposition in renal tissue leads to oxidative damage, fibrosis, and inflammatory responses [133, 134]. Therefore, mitigating tissue iron deposition seems to be more effective than ameliorating circulating iron deficiency in attenuating oxidative damage. The relationship between ferroptosis and CKD is currently not fully elucidated. Nonetheless, studies have established a tight correlation between ferroptosis and AKI, including AKI induced by rhabdomyolysis (RM) [135, 136], ischemia/reperfusion injury (IRI) [137–139], folic acid (FA) [140] and cisplatin [141]. It has been found that repressor element 1-silencing transcription factor (REST), a hypoxia regulator, participates in the transition from AKI to CKD, and tubule-specific REST knockout can significantly alleviate AKI and its progression to CKD by inhibiting ferroptosis [142]. Existing research suggests a bidirectional relationship between AKI and CKD, with AKI promoting CKD development, which, in turn, heightens susceptibility to AKI [141]. Besides AKI, the role of ferroptosis in various kidney diseases, including renal carcinoma, acute kidney injury, kidney fibrosis, and kidney inflammation, should not be overlooked [143]. Therefore, we believe that the role of ferroptosis in CKD is also substantial. CKD is a heterogeneous group of kidney diseases that includes several pathological types, such as diabetic nephropathy, hypertensive arteriolosclerosis, hereditary kidney disease, various glomerulonephritis and tubulointerstitial diseases (e.g., chronic interstitial nephritis, chronic pyelonephritis, uric acid nephropathy, and obstructive nephropathy). Renal fibrosis and glomerulosclerosis are two major pathological changes in CKD, with renal fibrosis, glomerulosclerosis, tubulointerstitial fibrosis, inflammatory cell infiltration and renal parenchymal loss collectively contributing to fibrosis progression.

### Diabetes kidney disease (DKD) and ferroptosis

DKD, the most prevalent microvascular complication of diabetes (DM), stands as a leading cause of ESRD, whose incidence reportedly increases with diabetes prevalence [144]. The pathogenesis of DKD is complex, and mounting evidence suggests that cell death is a direct factor affecting renal injury in DM [145]. Recent studies in animal models of DKD reveal the involvement of ferroptosis in its progression [146]. Wang et al. observed increased ACSL4 expression, decreased GPX4 expression, elevated lipid peroxides and iron content in a DKD mouse model. The ferroptosis inducers erastin and RSL3 were capable of inducing cell death in renal tubular cells in vitro [147]. Another study found that decreased expression of system Xc^−^ and GPX4 was noted in kidney biopsy samples from DM patients compared to non-DM patients. In TGF-β1-stimulated tubular cells, GSH decreased, and lipid peroxidation increased. Ferrostatin-1 (Fer-1) alleviated TGF-β-induced ferroptosis in DKD patients, suggesting that renal tubular cell death is related to ferroptosis and that repressing ferroptosis could potentially serve as a therapeutic strategy for DKD [148]. Li et al. also discovered a link between renal cell ferroptosis and high glucose conditions, with diabetic mice and human renal proximal tubular (HK-2) cells under high glucose conditions displaying iron overload, reduced antioxidant capacity, ROS accumulation, and lipid peroxidation. In the DKD model, treatment with Fer-1 significantly alleviated renal pathological damage. Their subsequent investigation revealed that Nrf2 levels decreased in DKD mice, but fenofibrate therapy could upregulate Nrf2, preventing ferroptosis [149]. Another study revealed that ZIP14 upregulated in a DKD model, while Fe^2+^ levels elevated, GPX4 and GSH levels reduced. Fe^2+^ and ZIP14 expression were both reduced by Fer-1 treatment [150]. However, it is worth mentioning that Fer-1 is unstable in vivo, which raises questions and challenges about whether Fer-1 should continue to be used in vivo research and follow-up therapy [151]. Andreas et al. discovered a new ferrostatin called 16–86, which is more metabolically stable in vivo than Fer-1 [114]. The identification of new ferrostatin may chart future directions in ferroptosis research. N-acetylcysteine (NAC), a nephroprotective agent, maintains mitochondrial redox homeostasis by enhancing mitochondrial GSH activity. In MDCK cells, NAC reduced high glucose-induced ferroptosis by increasing GPX4 expression [152]. Dapagliflozin, a glucose-lowering agent, has been shown to have cardiorenal protective effects, and recent studies have found that dapagliflozin also ameliorated ferroptosis in diabetic tubular injury by stabilizing SLC40A1 [153]. Empagliflozin also reduced renal tubular cell ferroptosis in DKD by enhancing the AMPK-mediated Nrf2 activation pathway [154]. All the above evidence indicates a strong association between DKD and ferroptosis, offering a new and meaningful treatment target for DKD.

### Hypertensive kidney damage and ferroptosis

Hypertension is a prominent etiological factor for CKD characterized by renal fibrosis. In patients with hypertensive renal impairment, lymphocytic infiltration, B-cell activation and IgG production have been found in the renal interstitium of damaged glomeruli and tubules [155]. Hyperhomocysteinemia (HHcy), affecting approximately 85% of CKD patients, is a risk factor associated with renal function decline [156]. HHcy is a reliable predictor of both renal function decline and the incidence of CKD in hypertensive patients [157]. It was previously reported that homocysteinemia (Hcy) upregulated oxidative stress and ferroptosis by enhancing GPX4 methylation [158]. A recent study unveiled that HHcy triggered the activation of B cells, leading to the production of pathogenic antiphospholipid binding protein β2GPI IgG, which was deposited in glomerular endothelial cells (GECs). This exacerbated glomerulosclerosis and impaired renal function, emphasizing that B-cell-derived β2GPI IgG produced by HHcy aggravates hypertensive renal damage by inducing ferroptosis in GECs. Modulating either the B-cell or ferroptosis pathway emerges as a promising therapeutic approach for hypertensive nephropathy [159]. By promoting the KLF15/Nrf2 signaling pathway, sirtuin7 (Sirt7) attenuated renal ferroptosis, lipid peroxidation, and epithelial mesenchymal transition (EMT) in the hypertensive state, thereby attenuating renal fibrosis [160]. The above evidence suggests that inhibiting ferroptosis appears to be a viable strategy to ameliorate hypertension-induced renal damage and renal fibrosis.

### Nephritis and ferroptosis

Immunoglobulin A nephropathy (IgAN) is the predominant primary glomerulonephritis and represents the leading cause of ESRD. Upon Gd-IgA1 stimulation in human mesangial cells (HMCs), a substantial reduction in peroxisome proliferator-activated receptor α (PPARα) and fatty acid-binding protein 1 (FABP1) levels was observed. Meanwhile, structural damage was observed in mitochondria, accompanied by increases in ROS and malondialdehyde (MDA), as well as decreases in GSH and GPX4. These results indicate that downregulation of the PPARa pathway reduces FABP1 expression, leading to alterations in GPX4 and ACSL4 levels that contribute to HMC ferroptosis and IgAN pathogenesis [161]. During oral Candida infection, the β-glucan receptor ephrin type-A 2 (EphA2) promotes disseminated candidiasis nephropathy and results in reduced renal inflammation and injury. Host cell ferroptosis could constrain antifungal effects. As such, ferroptosis represents a critical pathway in Candida-mediated immune pathology in the kidney [162]. Overproduction of ROS and abnormal infiltration of immune cells may be involved in lupus nephritis (LN) due to ferroptosis, as suggested by a bioinformatics study that identified eight ferroptosis-related genes (including *KRAS*, *Mapk14*, *PIK3CA*, *EGFR, SRC*, *mapk3*, *ATM* and *VEG*) that might be promising biomarkers of ferroptosis in LN [163]. Li et al. found that autoantibodies and interferon-a present in the serum of lupus susceptible mice or systemic lupus erythematosus (SLE) patients induce ferroptosis in neutrophils by enhancing the binding of the transcriptional repressor *CREMa* to the *GPX4* promoter. Neutrophil ferroptosis is an important driver of neutropenia in SLE [164]. Subsequent studies identified increased renal lipid peroxidation, increased ACSL4, decreased expression of SLC7A11, impaired GSH synthesis pathway, and diminished GPX4 expression in LN patients and mice. Ferroptosis predisposition in human proximal tubular cells was unveiled by sera from LN patients and mitigated by a novel ferroptosis inhibitor, liproxstatin-2 [165]. There is a robust association between ferroptosis and nephritis, particularly IgAN and LN. However, considering the diverse nature of nephritis, further investigations are warranted.

### Kidney transplantation and ferroptosis

The accumulation of senescent cells stands as a significant factor contributing to poor prognosis post-renal transplantation. While most existing antiaging drugs eliminate senescent cells through apoptosis induction, recent studies propose inducing senescent cell ferroptosis as an alternative strategy. Notably, a substantial portion of senescent cells post-transplantation are tubular cells [166]. Liao et al. demonstrated that murine renal tubular epithelial cells become susceptible to ferroptosis with aging, marked by increased expression of the proferroptotic lipoxygenase-5 (lox-5) and reduced expression of GPX4. Furthermore, they found that targeted elimination of senescent cells while sparing healthy tubular cells can be achieved through low-dose administration of the ferroptosis inducer RSL3 [167].

### Obstructive nephropathy and ferroptosis

Urinary tract obstruction stemming from various causes, including nephrolithiasis, ureteral malformations, prostatic hypertrophy, and neurogenic bladder, is a common contributor to CKD. Irisin emerges as an effective protective agent against obstructive kidney disease induced by unilateral ureteral obstruction (UUO). Irisin inhibits Smad3 phosphorylation-mediated ferroptosis and fibrosis, leading to significant alleviation of renal tubular cell injury and fibrotic lesions in UUO, as revealed by results [168]. Zhang et al. uncovered that ureteral obstruction induces ferroptosis in TECs in vivo. The ferroptosis suppressant, liproxstatin 1 (Lip-1), effectively reduced iron deposition and lipid peroxidation and reversed the low expression of GPX4 induced by UUO, ultimately suppressing ferroptosis in TECs. Lip-1 also attenuated profibrotic factor expression in the UUO model [169]. Furthermore, Fer-1 exhibited good performance alleviating oxidative-induced injury and fibrosis in renal tubular epithelial cells, as well as inhibiting calcium oxalate stone formation through the inhibition of ferroptosis [170]. This study also demonstrated the role of ferroptosis in obstructive nephropathy. With an increase in calcium oxalate concentration, the expression of p53, ACSL4 and TRC in renal tubular epithelial cells increased, while the expression of SLC7A11 and GPX4 decreased significantly, indicating the centrality of ferroptosis in CaOx crystal-induced renal injury [171]. A recent study further identified high expression of ACSL4 is highly expressed in a UUO mouse model, and the application of an ACSL4 inhibitor demonstrated a reduction in the interstitial fibrosis response [172].

### Other types of CKD and ferroptosis

In addition to the previously described prevalent types, various other causes contribute to CKD, and their research progress related to ferroptosis will be summarized. Autosomal dominant polycystic kidney disease (ADPKD) is an autosomal dominant genetic disorder in which kidney function is progressively impaired as more cystic fluid and larger cysts develop, and normal kidney tissue undergoes extrusion. Ferroptosis has been found to promote ADPKD progression. Deletion of the *Pkd1* gene, encoding polycystin-1, in mutant kidney cells and tissues of ADPKD affects the expression of key factors of ferroptosis [173]. Recent findings suggested that lipid peroxidation, associated with increased cyst volume, occurred via the chloride channel transmembrane protein 16A (TMEM16A) and cystic fibrosis transmembrane conductance regulator (CFTR). Transepithelial chloride secretion, promoting cyst enlargement in ADPKD, can be inhibited by GSH, CoQ10 and Fer-1, which reduce TMEM16A activation, subsequently diminishing cell proliferation and fluid secretion [174]. Bisphenol A (BPA), an industrial synthetic compound, induces acute and chronic kidney injury and exacerbates various kidney conditions. Bao et al. observed that BPA increase iron accumulation and lipid peroxidation in renal tubular epithelial cells, leading to ferroptosis. The use of Fer-1 and deferoxamine significantly reduces cell death [175]. Long-term administration of aristolactam I (ALI)-containing drugs is associated with aristolochic acid nephropathy (AAN). Intriguingly, ALI dose-dependently suppresses the protein content of Nrf2, HO-1, and GPX4. The Nrf2-HO-1/GPX4 antioxidant system emerges as a potential intervention target for preventing ALI-containing drug-induced nephropathy [176]. Guan et al. identified two genes, charged multivesicular body protein 1a (CHMP1A) and dipeptidase 1 (DPEP1), associated with kidney disease that could regulate ferroptosis by altering cellular iron transport [141].

### Cross-talk between ferroptosis and different modes of cell death in CKD

Cell death pathways were previously thought to be parallel and independent of each other. However, recent studies have found that there may be mutually regulated pathways between different forms of cell death. Caspase-8, initially thought to be an apoptosis effector, was found to be a central regulator of cell death, promoting apoptosis, necroptosis, or pyroptosis depending on its posttranslational state and cell type [177]. There is evidence that the same regulator can regulate ferroptosis and other forms of cell death through different pathways. Abnormal activation of the NOD-like receptor family pyrin domain-containing 3 (NLRP3) inflammasome has been associated with the onset of several inflammatory diseases, promoting various types of cell death, including apoptosis, necroptosis, and ferroptosis [178]. Crucial molecules of ferroptosis, GPX4 and Nrf2, have been found to inhibit the activation of the NLRP3 inflammasome [179, 180]. Additionally, RIPK3 and caspase-8 recruitment have been shown to activate the NLRP3 inflammasome, triggering necroptosis and pyroptosis [181]. NLRP3 inflammasome activation in podocytes was considered a prerequisite for DKD [182]. And NLRP3 inhibitors can significantly improve proteinuria and renal function in mice, underscoring the importance of the NLRP3 inflammasome in kidney disease [183, 184]. Although most studies have emphasized the association of NLRP3 with autophagy, considering its role in other cell death pathways necessitates further exploration. In addition, previous studies have identified p53 as a regulator of apoptosis and autophagy. Later, p53 acts as a transcriptional inhibitor of SLC7A11, inhibiting Cys intake and promoting ferroptosis [185]. Zhang et al. simulated iron overload during the progression of Parkinson’s disease, revealing that ferroptosis occurred first in the low-concentration iron treatment group, followed by apoptosis with increased occurred after the increase in iron dose. Ferroptosis inhibitors can rescue apoptosis caused by iron overload, whereas apoptosis inhibitors cannot prevent death, and the underlying mechanism may be related to the p53 signaling pathway [186]. Studies further confirmed that p53 served as an autophagy regulator to alleviate kidney injury [187]. In mouse models, the MDM2-p53-LMNB1 axis also prevented mitochondrial damage and ferroptosis in metanephric tubular epithelial cells in AKI [188]. BECN1 expression can promote apoptosis and autophagy and affect the activity of system Xc^−^, inducing ferroptosis [80, 189]. Several autophagy-related genes have been identified as positive regulators of ferroptosis, and blocking autophagy can inhibit the accumulation of ROS and ferroptosis [76]. Different forms of cell death are closely related, and there are overlapping molecular regulatory pathways between ferroptosis and other cell death modes, such as necroptosis, apoptosis, pyroptosis and autophagy, collectively playing a role in CKD. However, future research faces the challenge of exploring how different types of cell death interact, given that most studies have predominantly focused on a single form of cell death.

### Outlook

With the rapid advancement of the economy and society, coupled with continuous improvements in living standards, the prevalence of metabolic disorders like obesity, diabetes, hypertension, hyperlipidemia and hyperuricemia, is on the rise. Correspondingly, the burden of kidney disease is increasing, and CKD is projected to become one of the top five causes of death by 2040 [190, 191]. The escalating number of CKD patients and the limited treatment strategies pose an urgent need to unravel CKD pathogenesis and explore new therapeutic avenues for CKD. In this comprehensive review, we provide a concise summary elucidating key mechanisms implicated in the development and progression of ferroptosis. These encompass disturbances in iron metabolism, compromised antioxidant capacity, lipid peroxide accumulation, and mitochondrial dysfunction. Subsequently, we discussed the progress of ferroptosis research in CKD, aiming to provide new therapeutic strategies for CKD patients.

Over the past decade, there has been a remarkable surge in ferroptosis research. In general, it is an iron-dependent form of cell death mediated by lipid peroxidation and regulated through multiple pathways. The increasing number of studies on the role of ferroptosis in kidney disease has unraveled its involvement in tubular injury and renal fibrosis. Ferroptosis has been identified as one of the pathways contributing to renal fibrosis in vivo, and pharmacological targeting of ferroptosis shows promise in driving cells toward adaptive repair and ameliorating fibrosis [21]. However, the existing studies on the connection between ferroptosis and disease have focused on specific indicators, such as GSH, GPX4, ROS, and Nrf2 levels. While these studies confirm the existence of ferroptosis and oxidative accumulation, the specific and complex mechanism remains to be fully elucidated. With various forms of cell death observed in CKD, such as necroptosis, apoptosis, and autophagy, the question of whether ferroptosis predominates in kidney disease and whether multiple modes of cell death interact with each other needs to be emphasized and addressed. Additionally, understanding how certain drugs or chemicals modulate renal fibrosis by affecting ferroptosis demands further investigation. Interactions between ferroptosis and other forms of cell death exist, prompting questions about whether inhibiting ferroptosis interferes with other cell death pathways. Despite these challenges, ferroptosis offers novel perspectives on CKD research and treatment.

Ferroptosis is a vast network, and while multiple studies have demonstrated a connection between ferroptosis and CKD, current research primarily focuses on classical ferroptosis regulatory molecules. The complete pathway requires further exploration. As ferroptosis is a recently identified form of cell death, research has predominantly employed animal models, and clinical studies targeting ferroptosis in CKD are limited. Nevertheless, the undeniable potential of ferroptosis suggests it could offer a novel and efficacious therapeutic avenue for CKD treatment. Therefore, this review aims to summarize current research progress on ferroptosis and its pivotal role in CKD, providing insights for subsequent studies to establish ferroptosis as a therapeutic target for CKD.
